# Food-Grade Physically Unclonable Functions

**DOI:** 10.1021/acsami.3c09035

**Published:** 2023-08-24

**Authors:** Abidin Esidir, Nilgun Kayaci, N. Burak Kiremitler, Mustafa Kalay, Furkan Sahin, Gulay Sezer, Murat Kaya, M. Serdar Onses

**Affiliations:** † ERNAM—Nanotechnology Research and Application Center, Erciyes University, Kayseri 38039, Turkey; ‡ Department of Materials Science and Engineering, Erciyes University, Kayseri 38039, Turkey; § Department of Electricity and Energy, Kayseri University, Kayseri 38039, Turkey; ∥ Department of Biomedical Engineering, Faculty of Engineering and Architecture, Beykent University, İstanbul 34398, Turkey; ⊥ Department of Pharmacology, Erciyes University, Faculty of Medicine, Kayseri 38039, Turkey; # Department of Molecular Biology and Genetics, Faculty of Science and Letters, Istanbul Technical University, Istanbul 34469, Turkey

**Keywords:** physically unclonable function (PUF), corn starch, edible, fluorescence, encoding

## Abstract

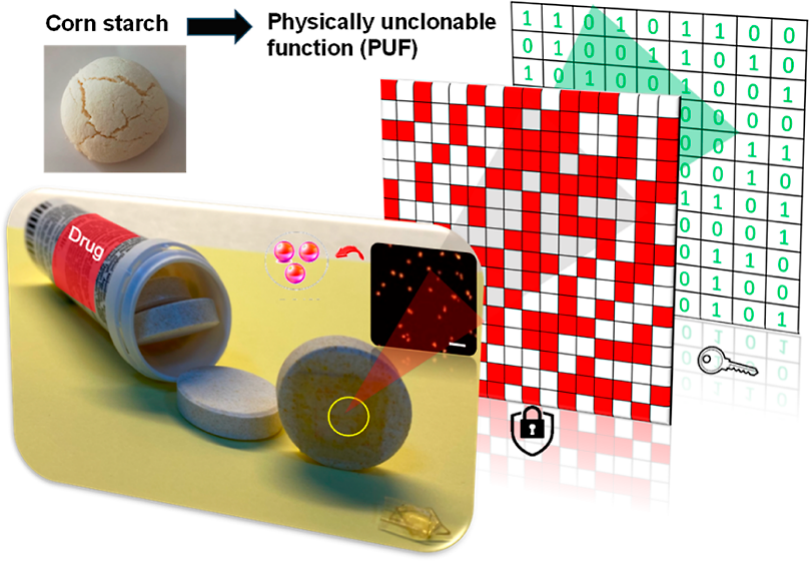

Counterfeit products in the pharmaceutical and food industries have posed an overwhelmingly increasing threat to the health of individuals and societies. An effective approach to prevent counterfeiting is the attachment of security labels directly on drugs and food products. This approach requires the development of security labels composed of safely digestible materials. In this study, we present the fabrication of security labels entirely based on the use of food-grade materials. The key idea proposed in this study is the exploitation of food-grade corn starch (CS) as an encoding material based on the microscopic dimensions, particulate structure, and adsorbent characteristics. The strong adsorption of a food colorant, erythrosine B (ErB), onto CS results in fluorescent CS@ErB microparticles. Randomly positioned CS@ErB particles can be obtained simply by spin-coating from aqueous solutions of tuned concentrations followed by transfer to an edible gelatin film. The optical and fluorescence microscopy images of randomly positioned particles are then used to construct keys for a physically unclonable function (PUF)-based security label. The performance of PUFs evaluated by uniformity, uniqueness, and randomness analysis demonstrates the strong promise of this platform. The biocompatibility of the fabricated PUFs is confirmed with assays using murine fibroblast cells. The extremely low-cost and sustainable security primitives fabricated from off-the-shelf food materials offer new routes in the fight against counterfeiting.

## Introduction

Counterfeiting has been an increasingly serious concern with substantial economic, social, and health consequences. Counterfeit products can be found in almost all industries, from everyday textiles to advanced electronic devices. In consideration of direct health effects, counterfeit medications and food are of prime significance. The measures against such counterfeiting should be prioritized since they are consumed by the digestive or circulatory systems.^1^ To give just one tragic example, it is estimated that 250,000 children die each year as a direct result of using counterfeit malaria and pneumonia medications.^2^ Food fraud is also a significant threat to society. According to the 2019 annual report by the European Union Food Fraud Network, types of food fraud include mislabeling, the replacement and dilution of ingredients, manipulated documentation, and the use of unapproved processes. The consumer consciousness of natural and authentic food products and dietary supplements together with the rise of online shopping further motivates the development of sustainable approaches to fight against counterfeiting.

Conventional anticounterfeiting methods focus on attachment of security labels to the packaging and include watermarks, holograms, graphical barcodes, security inks, and radio frequency identification tags.^3–5^ Repackaging counterfeit products can easily circumvent these security measures. A promising solution to this problem is direct attachment of security labels on the actual product. Commonly referred to as “on-drug” or “on-dose” authentication, it has attracted significant interest in the fight against counterfeit medicine. Pharmaceutical ingredients,^6–9^ polymer/hydrogel microstructures,^10–12^ silicon nanoparticles,^10^ DNA tags,^13^ and transgenic silk-based labels^14^ have been used to fabricate on-drug security labels. Despite these advancements in the aforementioned drug authentication methods, there are still inherent problems that should be remedied. Some of these techniques offer a high degree of security based on functional materials in their content and the unique characteristics of these materials against particular stimuli or due to their specialized architecture. The manufacturing of security labels using a deterministic method implies that third parties with access to the same materials and production equipment can potentially reproduce them. Almost all of the materials used in these methods are not food-grade, and there are concerns about taking them as oral intake due to doubts in terms of long-term toxicological consequences.

According to our standpoint, the ideal on-drug and on-food security approach should meet three criteria: (i) unbreakable encoding; (ii) constructed from food-grade materials; and (iii) low-cost materials and scalable processes. The first requirement can be fulfilled by adopting a physically unclonable function (PUF) based approach. PUFs use physical systems rather than mathematical functions and are based on the concept of challenge–response pairs. The physical system responds to a challenge in a way that is both distinctive and impossible to regenerate. The stochastic physical process that is used to generate PUFs is what gives them their one-of-a-kind features and prevents them from being cloned.^15–17^ PUFs are frequently created by using a method that makes use of randomization in the spatial placement of cryptographic primitives. Since the pioneering work,^15^ optical PUF systems have been an active study area. Over the past decade, a variety of material types and production methods have been investigated in an attempt to demonstrate optical PUFs for various application areas, including randomly positioned quantum dots,^18,19^ perovskite nanocrystals,^20,21^ plasmonic nanoparticles,^22–25^ 2D materials,^26^ spontaneously wrinkling-folding based synthetic materials,^26–28^ dewetting thin films of polymers^29^ and light-emitting organic molecules,^24,30^ fluorescent biomaterials,^8,31^ carbon-based materials,^32,33^ and electrosprayed polymeric particles.^34^ Despite these, it is fair to claim that for pharmaceutical and food applications, PUF systems that are amenable to the on-product approach have not yet been introduced. According to our knowledge, the only exceptional effort in this field is the recently presented on-drug edible PUFs using fluorescent transgenic silks by Kim et al.^31^ Therefore, in order to fill the huge gap in this field, there is a great need for PUF systems that can be created from entirely food-grade and inexpensive components by using practical fabrication techniques.

Here, we demonstrate the simple yet highly effective fabrication of PUFs using food-grade corn starch (CS) microparticles. The essential idea proposed in this study is that food-grade CS particles exhibit highly appealing characteristics as encoding elements for edible PUF applications. These characteristics include microscopic dimensions, particulate structure, ability to adsorb taggants, low-cost, widespread availability, and edibility. CS is widely used as a natural ingredient in drugs and food products, making it a very attractive material for on-drug or on-food security label applications. CS is composed of microscopic particles with a broad distribution of size and morphology. This type of polydispersity facilitates randomness, even in simple manufacturing processes. Because of its large surface area and favorable surface chemistry, CS is a well-known adsorbent material. Highly active exchangeable hydroxyl groups in the structural unit promote interaction with various functional groups.^35–39^ This characteristic makes CS appealing to adsorb taggant molecules with fluorescence, Raman scattering, and other unique properties. This approach assists in generating surfaces with unique and specific responses. Based on these peculiarities, this work proposes food-grade PUFs made from CS microparticles modified with erythrosine B (CS@ErB). ErB is an organoiodine compound that is widely used for coloring food. While the unclonable surface with intrinsic stochasticity was created by a straightforward spin-coating technique using random positioning of CS@ErB particles, the distinct fluorescence profile and chemical structure of ErB formed an additional security layer. Unclonable CS@ErB features were transferred onto films of food-grade gelatin, which can be placed onto medications and foods. As an eventual outcome, the first successful demonstration of the generation of optical PUFs from food-grade materials is presented in the form of edible security labels with nearly ideal figures of merit.

## Experimental Section

### Materials

Silicon wafers were purchased from University Wafer. Commercially available bovine gelatin (Dr. Oetker) and CS (Kent) were used in the experiments. Erythrosine B, known as E127, was purchased from Neelikon. Purified water was used throughout the experiments.

### Preparation of CS@ErB Particles

The CS particles (0.01 g/mL) were homogeneously dispersed with an ultrasonic probe for 20 min. ErB was added to the CS dispersion at a concentration of 5 mM. To see the ability of CS particles to adsorb ErB, the washing process was carried out with water. CS@ErB particles were washed by centrifugation at 4000 rpm for 10 min and redispersed in water. To completely remove unadsorbed ErB molecules, this washing process was repeated three times. The CS@ErB particles were dried at 50 °C for 5 h in a vacuum oven.

### Fabrication of PUFs

The first step consisted of the preparation of a gelatin substrate. For this purpose, 10 g of gelatin and 60 mL of water were mixed in a beaker. The dissolution process was carried out on a hot plate at 100 °C for 30 min under continuous agitation with a magnetic stirrer. The gelatin solution was poured into Petri dishes (8 mL for each dish) and left to solidify at room temperature. 50 μL of the CS@ErB colloidal solution was spin-coated on a silicon substrate for 30 s at 4000 rpm. The randomly positioned CS@ErB particles were transferred onto the previously prepared 1 × 1 cm^2^ gelatin substrate with a thickness of ∼500 μm. The transfer process was facilitated by placing the inverted silicon substrate with randomly positioned CS@ErB particles on the gelatin. A load of 100 g (9.8 kPa for a 1 × 1 cm^2^ substrate) was placed on top of the inverted silicon substrate for 5 s to ensure the transfer of CS@ErB particles from the donor silicon substrate to the receiver gelatin substrate.

### Characterization

An upright research microscope (ZEISS Axio Imager 2) was used to acquire optical and fluorescence images of the samples. For fluorescence microscopy imaging, a 100 W mercury arc lamp (HBO 100) was used as the multispectral light source. The fluorescence images were acquired by using filter set 20 (BP546/12 excitation filter and BP575-640 emission filter) with an FT560 beam splitter. The images were obtained with 10× and 20× objectives. A hand-held microscope (Dino-Lite AM7115MT-FUW) with a UV light source of 375 nm was used to demonstrate authentication with compact tools. The morphology of the features was analyzed by scanning electron microscopy (SEM) (Zeiss EVO LS10) operated at 25 kV. Raman spectra and mapping images were obtained with a confocal Raman spectrometer (WITec Alpha300 M+) integrated with a fine-focusing microscope. The wavelength and power of the laser were 532 nm and 0.1 mW, respectively. Infrared spectra were obtained using a PerkinElmer 400 Fourier transform infrared (FT-IR) spectrometer with a MIRacle attenuated total reflection accessory. UV–vis spectroscopy (PerkinElmer Lambda 25) was used to monitor the presence of ErB. X-ray diffraction (XRD) measurements were made via an X-ray diffractometer (Bruker AXS D8) using a Cu–Kα source at a scanning step of 0.1° for 2θ range of 2–40°. Thermal gravimetric analysis was performed in a PerkinElmer Diamond system up to a temperature of 600 °C in a nitrogen atmosphere with a heating rate of 10 °C min^–1^ from the ambient temperature. Photoluminescence and absorbance measurements were carried out by using a fluorescence spectrophotometer (Agilent-Cary Eclipse) and a UV–vis spectrometer (Thermo Genesys 10S), respectively. The photoluminescence quantum yield of ErB was calculated by taking rhodamine 123 as the reference.

### Cytotoxicity Test

According to the ISO10993-5-2009 standard, the L929 cell line (murine fibroblast, American Type Culture Collection; ATCC, Manassas, VA) was selected for the evaluation of the cytotoxicity of the material. The cell viability was evaluated with two different techniques: MTT and fluorescence live/dead cell assays. Different weights (50, 10, and 1 mg) of gelatin substrates with CS@ErB and CS particles were sterilized under UV light for 40 min. 1 mL of complete medium (Dulbecco’s modified Eagle’s medium; DMEM, Sigma Chemical Company, USA) supplemented with 10% fetal bovine serum (Gibco BRL, USA), 1% penicillin/streptomycin (Sigma-Aldrich, Germany), and 1% glutamine (Gibco, UK) was added to each of the sterilized samples. After 10 min of gentle vortexing, the materials were dispersed in the medium. The cell culture medium was filtered through a 20 μm mesh filter to remove possible insoluble microscopic agglomerations in the suspension. L929 cells with a density of 6 × 10^3^ cells/wellwere then plated in 96-well culture plates with this suspension of cell culture medium/PUFs at an initial density of 6 × 10^3^ cells/well. L929 cells (6×103 cells/well) were seeded in triplicate in 96-well plates, incubated overnight, and then treated with this suspension of cell culture medium/PUFs (1,10 or 50 mg/ml) for 24 h.The number of cells seeded in wells was determined by counting from a 0.4% trypan blue-stained cell suspension on a Thoma slide. An equal starting number of L929 cells incubated simultaneously and under the same conditions as the PUF untreated cell culture medium was considered as controls. After incubation, the overall morphology of the cells was evaluated by means of an optical microscope. Then, the cells were incubated with 10 μL of 3-(4,5-dimethylthiazol-2-yl)-2,5-diphenyltetrazolium bromide solution (MTT, 5 mg/mL, Sigma-Aldrich, Germany) for 3 h to analyze viability. After the incubation, formazan crystals were dissolved in 100 μL of dimethyl sulfoxide, and absorbance values at 560 nm were measured with the aid of a microplate reader. Cell viability was calculated by using the following equation
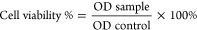


The mean OD values were normalized compared to the control group and calculated as the cell viability (%). Each result is given as the average of the studies performed in three replicates at three different times.

For the fluorescence live/dead cell assay [SYTO9 and propidium iodide (PI), Thermo Fisher Scientific], trypsin solution was added to the cell culture incubated with suspension of cell culture medium/PUFs under the same conditions mentioned above, and cells were removed from the well surfaces. A complete medium was then added to inactivate trypsin, and cells were suspended. The resulting cell suspension was transferred to an Eppendorf tube. It was gently centrifuged for 5 min at 300*g*, and the supernatant was discarded. The cell pellet was gently resuspended in a 0.85% NaCl solution. This washing process was repeated three times to thoroughly remove the media residues in the suspension. Then, 3 μL of SYTO 9 (diluted to 1 mM in water) and 1 μL of PI (diluted to 2 mM in water) were added to 100 μL of cell suspension (2 × 10^5^ cells) and incubated for 15 min at room temperature in the dark. After incubation, 10 μL of the stained cell suspension was pipetted onto a glass slide, and fluorescence images were taken using a ZEISS Axio Imager 2 microscope. Live and dead cells were counted using ImageJ.

### PUF Performance Analysis

The analysis was performed using fluorescence microscope images. PUF parameters and binary keys were generated with code written in MATLAB software. First, fluorescence microscope images (2752 pixels × 2208 pixels) were converted from the RGB color space to the LAB color space. Second, fluorescence microscopy images converted to grayscale were processed using inversion, dehazing, and noise reduction algorithms. Von Neumann debiasing was applied, and 256 (16 × 16) bit-long PUF keys were obtained by converting the original images to binary codes. Red and white bits refer to 0 bits and 1 bits, respectively. Uniformity, uniqueness, and *p*-values were calculated from these binary security keys using MATLAB software.

### Direct Authentication via Feature Detection Algorithm

The authentication involved the utilization of the Oriented FAST and Rotated BRIEF (ORB) algorithm implemented in MATLAB. The “detectORBFeatures” function was used to extract image features, which were then matched with features from the database using a nearest-neighbor-based approach. To address mismatches, a random sample consensus (RANSAC) algorithm was applied. Recognition scores were computed based on the successful matching of feature points.

### Stability Experiments

The photostability test was carried out with an exposure time of 600 ms. 100 fluorescence microscope images were taken consecutively with 1 min intervals from the same area of the sample. The ZEN Blue Lite software of the ZEISS Axio Imager 2 microscope was used for the fluorescence intensity measurements of randomly generated PUF features. For UV stability, the surface was exposed to UV light with a power of 3 W at a wavelength of 365 nm for 6 h. The mechanical stability of PUFs was studied with a linear abrasion test. Specifically, the sample was glued under a weight of 200 g and moved 30 cm against aluminum foil.

## Results and Discussion

### Fabrication of Food-Grade CS@ErB-Based PUFs

**Figure 1:**
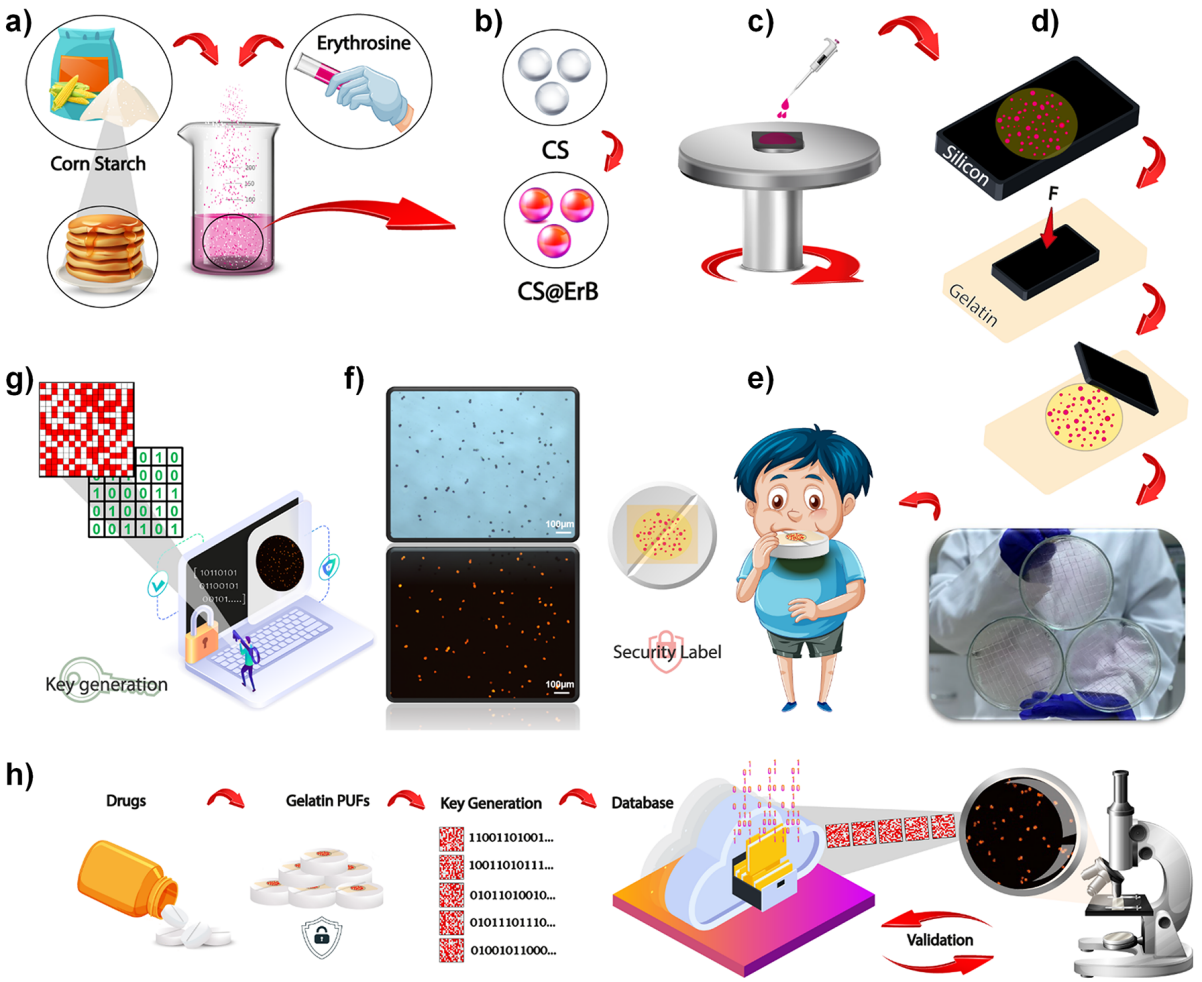
Generation of PUFs using all food-grade materials. (a) Homogeneous dispersion of CS particles and ErB was prepared in water with an ultrasonic probe. (b) Adsorption of ErB by starch particles (CS@ErB). (c) Spin-coating of the colloidal dispersion of CS@ErB. (d) Transferring randomly positioned CS@ErB features from the silicon wafer to gelatin. The photograph at the bottom shows 3 large-area PUFs. Each sample was fabricated by the transfer of randomly positioned CS@ErB features from the 4 in. silicon wafer to gelatin. (e) Application demonstration on a drug tablet. (f) Optical and fluorescence microscope images of PUFs. (g) Generation of binary keys from fluorescence microscope images. (h) Schematic illustration of the authentication process.

Figure 1a shows the fabrication of PUFs entirely based on food-grade materials. At the center of this approach is starch, which is produced from corn and is widely available for baking. CS was used directly without any purification or additional processing steps. CS consists of microscale particles of carbohydrates. The random dispersion of these particles enables the generation of PUFs. Such particles are visible under visible light illumination. The irregular size and geometry of starch particles together with their random distribution make duplication nearly impossible, even under bright field microscopy. The duplication can be further challenged by coupling taggant molecules by benefiting from the adsorbent nature of CS. Taggants of varying molecular structures can be designed and authenticated by means of fluorescence microscopy, Raman spectroscopy, and other techniques. For the purpose of fabricating all food-safe PUFs, we selected a food-grade fluorescence taggant, ErB, in this study. The excitation and emission wavelengths of ErB were measured as 528 and 534 nm based on the photoluminescence and absorbance spectra (Figure S1), respectively. The photoluminescence quantum yield of ErB was 1.4%. Thanks to the superior adsorbent properties of CS, ErB was strongly adsorbed to the starch particles. Following removal of excess ErB, the resulting CS@ErB particles were deposited on a silicon wafer by spin-coating (Figure S2). During the spin-coating process, ErB-adsorbed CS particles were randomly dispersed over the surface. The random positions of the particles provide the stochastically defined encoding layer. To generate a completely food-grade security label, CS@ErB was then transferred onto the surface of gelatin (Figure 1d). For the transfer process, the donor silicon substrate with random features was turned upside down and placed on the acceptor gelatin substrate. As depicted in Figure S3, a significant portion of CS@ErB particles on the donor substrate can be transferred onto the gelatin during this process. The presented approach can be easily scaled up, thanks to the use of low-cost and abundant materials. Figure 1d presents the large-area fabrication of PUFs using a 4 in. silicon wafer as the donor substrate (see Figure S4 for additional images). The resulting PUFs can be simply cut into small pieces and attached to goods. For demonstration purposes, the gelatin with CS@ErB particles was integrated with a drug tablet by lightly moistening the back of the gelatin substrates with a paintbrush and pressing them onto the drug surface (Figure 1e, also see Video S1). Optical and fluorescence microscopy images (Figure 1f) show randomly positioned CS@ErB particles. Fluorescence microscopy was used for image analysis of randomly positioned features on gelatin by utilizing the photoluminescence of ErB. The presented approach is adaptable to compact microscopes. Figure S5 presents fluorescence images of randomly positioned CS@ErB particles obtained via a hand-held microscope. Authentication of PUFs is achieved by converting images to binary keys consisting of 0 and 1 bits (Figure 1g). In a practical setting, these keys are stored in a database. To authenticate the label, a fluorescence image is taken by the user, and the binary keys obtained from the image are compared with the ones in the database (Figure 1h). Alternatively, a direct comparison of fluorescence images can be performed by benefiting from feature-matching algorithms. Manufacturers and end users can ensure the authenticity and traceability of the products throughout the supply chain.

The preparation and characterization of the CS@ErB particles were studied first. CS has favorable adsorbent properties.^40–42^ This characteristic allows for easy adsorption of fluorescent dyes. The starch particles were dispersed in a ratio of 1:100 (w/w) in water, and then ErB solution (5 mM) was added to yield CS@ErB. A uniform dispersion of starch particles loaded with ErB was obtained in this manner. To remove excess ErB molecules, the dispersion was centrifuged and redispersed in water three times. The washed CS@ErB was then used in the further characterization and manufacturing of PUFs.

### Characterization of Food-Grade CS@ErB-Based PUFs

**Figure 2:**
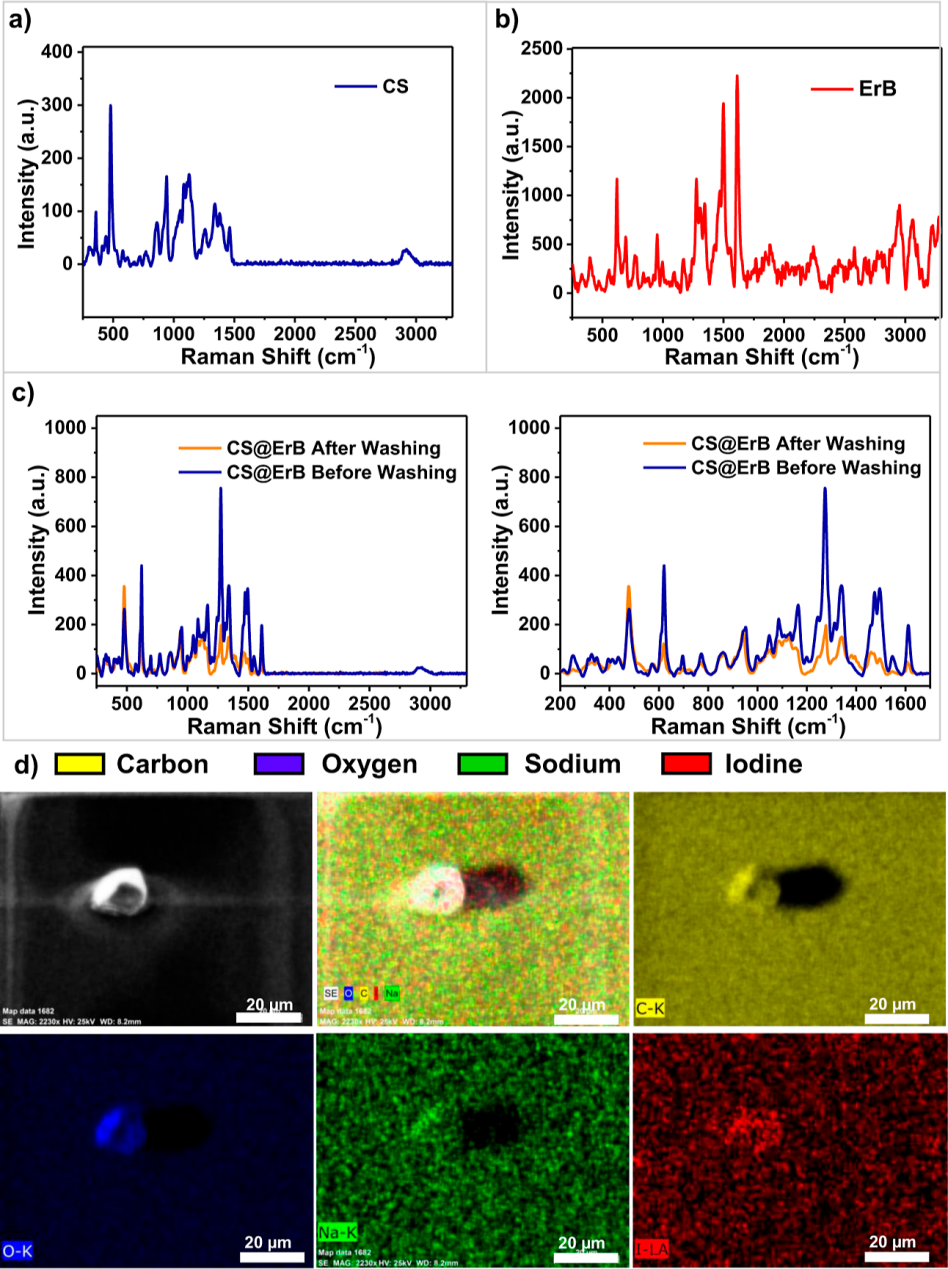
Raman spectra of (a) CS, (b) ErB, (c) CS@ErB, and (d) SEM and EDX mapping images of CS@ErB transferred to gelatin.

The chemical characterization of the CS@ErB particles was performed with FT-IR and Raman spectroscopy. Figure 2 presents Raman spectra for CS, ErB, and CS@ErB materials. In the Raman spectrum of CS, all the peaks at 477, 767, 1130, and 1339 cm^–1^ are in close agreement with the literature (Figure 2a). The peak observed in the region of 477 cm^–1^ originates from C–C–C and C–O bonds, whereas the peak around 767 cm^–1^ is related to the C–C–O bond. The peaks positioned at 1130 and 1339 cm^–1^ refer to C–O, C–C, and C–O–H bonds. The peak around 2900 cm^–1^ corresponds to the symmetric and asymmetric CH stretching in the starch.^43^ The Raman spectrum of ErB exhibited asymmetric stretching of the –COO group positioned at around 1610 cm^–1^. The strong peak at around 1500 cm^–1^ belongs to the C–H deformation in the benzene ring. The peaks at 1470 and 1344 cm^–1^ are caused by in-plane C–H and C–C deformations of the xanthene ring. The peak at 1270 cm^–1^ belongs to the asymmetric stretching mode of the C–O–C and benzene ring and the main xanthene ring in the structure (Figure 2b).^44^ In the Raman spectrum of CS@ErB, ErB and CS peaks were observed together, while after repeated washing of CS@ErB, the intensity of the ErB peaks decreased with the removal of excess dye molecules. In addition, the Raman mapping image of the CS@ErB particles was taken according to the specific band of ErB at 1612 cm^–1^ (Figure S6). Due to the adsorption of ErB on starch, the specific band of ErB shifted to 1610 cm^–1^ in the Raman spectrum of the CS@ErB particle. With both the spectrum and the mapping image, we show that ErB was successfully adsorbed to CS (Figure 2c). SEM and EDX mapping images of the randomly dispersed CS@ErB particles further support the proposed adsorption process. C and O elements in the chemical structure of both CS and ErB were shown in the EDX mapping. In addition, the presence of I and Na that are only found in ErB, which clearly demonstrates the adsorption of dye onto CS particles (see EDX spectrum in Supporting Information, Figure S7).

**Figure 3:**
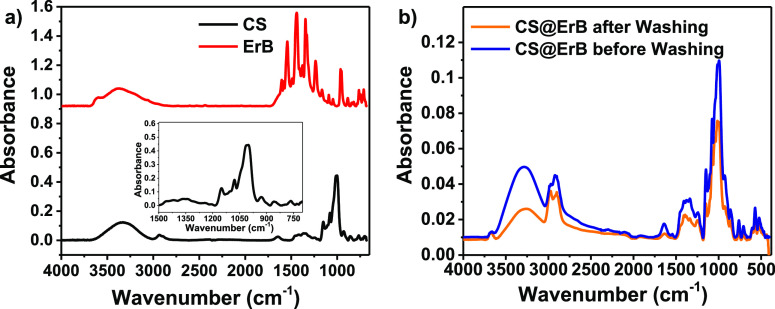
FT-IR spectra of (a) ErB and CS and (b) CS@ErB before and after washing.

Figure 3 presents FT-IR spectra of ErB, CS, and CS@ErB. FT-IR spectra of ErB and CS exhibited characteristic bands of the individual materials. The FT-IR spectrum of the CS@ErB material confirmed the successful adsorption of ErB over CS. For ErB, the peak observed at 1233 cm^–1^ is due to –C–OH stretching, and the broad peak observed at 3200 to 3500 cm^–1^ is due to –OH stretching. The peaks at 1609, 1541, 1342, and 1435 cm^–1^ belong to the stresses in the benzene ring in the structure of ErB. The peak at 951 cm^–1^ is related to the –C=C–H functional group in the molecular structure of ErB (Figure 3a).^45,46^ The broad peak at 3335 cm^–1^ and the sharp peak at 2938 cm^–1^ refer to –OH and –CH_2_ (axial deformation) groups, respectively. The peak at around 1355 cm^–1^ is caused by the –C–OH bending vibration. The peaks between 1150 and 760 cm^–1^ are stretching vibrations of C–O–H, C–O–C, and C–O bonds in the anhydroglucose ring of the CS (Figure 3a).^47,48^ Specific peaks of both CS and ErB were observed for the CS@ErB particles. The peaks around 3300 and 2900 cm^–1^ of CS are due to –OH and –CH_2_ groups, while the ones between 1150 and 760 cm^–1^ are caused by the C–O groups in the structure with small shifts, which were likely caused by the adsorption of ErB onto CS. A decrease in the intensity of the ErB peaks is observed in the spectrum of the CS@ErB particles. This decrease is attributed to removal of the excess and unadsorbed ErB (Figure 3b).

The crystallographic and thermal characterization of the CS@ErB further proved the successful preparation of the material. XRD patterns of CS, ErB, and CS@ErB particles were obtained from powdered samples (Figure S8a). The diffraction pattern of CS exhibited characteristic peaks at 15.1, 17.7, 20.1, and 23.2°, consistent with the literature.^48^ The small peak observed at 10° in the XRD spectrum of CS is attributed to amylopectin, one of the main components of starch.^49^ The diffraction pattern of ErB matched previous studies,^50^ showing diffraction planes at 22.6, 27.3, and 31.6°. The CS@ErB particles displayed a diffraction pattern similar to that of CS, with the low adsorption of ErB making it difficult to identify specific peaks. Thermal analysis (Figure S8b) revealed moisture removal below 100 °C and a significant mass loss at around 313 °C for CS and CS@ErB, likely due to polysaccharide degradation. ErB experienced the largest mass loss at around 380–410 °C and 40% of the mass was lost at 600 °C in good consistency with the literature.^51^ Further details regarding characterization are presented in the Supporting Information.

### Biocompatibility of PUFs

**Figure 4:**
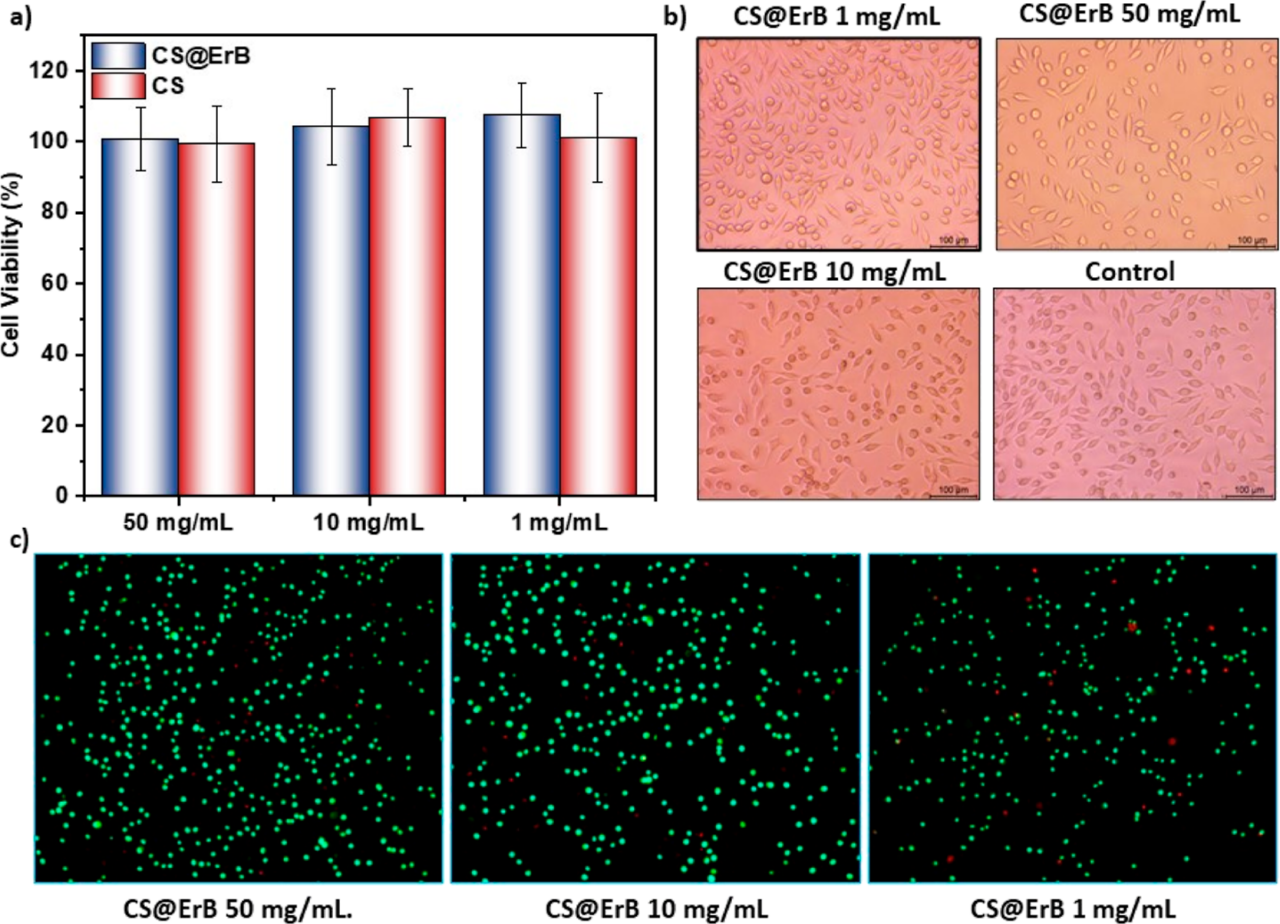
Biocompatibility of PUFs constructed from CS@ErB particles. (a) Cell viability results by the MTT assay. (b) Optical microscopy images of cells incubated with different concentrations of CS@ErB. (c) Imaging of live and dead cells under a fluorescence microscope. Green-fluorescent cells are alive, while red cells are dead. CS@ErB and CS particles on a gelatin substrate were used for the biocompatibility experiments.

The proposed PUFs are constructed from food-grade materials. CS, ErB, and gelatin can be safely digested and biocompatible at an individual level. To confirm the biocompatibility of PUFs made from these materials and ensure any toxicity that may arise from the interaction of these materials, we used two different assays. Figure 4 presents the in vitro biocompatibility of PUFs based on CS@ErB particles. The biocompatibility experiments were performed on CS@ErB and CS particles deposited on gelatin substrates using the MTT assay, which is considered as the gold standard^52^ of cell viability tests. The MTT assay provided an expression of the mitochondrial NADPH metabolic activation of cells incubated with the material and yielded results directly related to the viability of the cells. Accordingly, even at very high concentrations, cells cultured with the sample (CS@ErB) had an average viability of 100.8 ± 8.9(%) compared to cells cultured with a complete medium. Moreover, CS and CS@ErB both yielded similar cell viability results, which can be considered a clear indication that the combinations of food-grade materials used do not impair cellular function. The morphological structures of the cells can also provide observational data on apoptotic cells. As seen in Figure 4b, cells incubated with CS@ErB and control cells have similar morphology. This observational result is another indication that the material does not induce apoptotic cell death and is biocompatible. On the other hand, both MTT and optical images do not provide clear information about cellular damage that does not cause cell death. To further evaluate this type of damage, cells were stained with PI, which emits red fluorescence and only penetrates dead or damaged cells.^53^ The cells were also stained with SYTO9, which emits green fluorescence and binds to nucleic acids of eukaryotic/prokaryotic cells. As seen in Figures 4c and S9, damaged or dead cells are very few, with a fraction of 6.1% when 50 mg/mL CS@ErB is used. In other words, the proposed platform does not cause any cellular damage in 94 of 100 cells. Here, cellular damage was also observed at a rate of 5.8% in the control group. This result shows that there is no cellular damage caused by CS@ErB. In summary, a detailed analysis of in vitro cytotoxicity of PUFs developed with renewable materials has proven the high biocompatibility of the proposed platform. However, ErB can cause toxicity under light by triggering the generation of reactive oxygen species and uncontrolled oxidation.^54^ To probe this type of toxicity, a biocompatibility test was performed under exposure to light. CS@ErB has a biocompatibility close to 100% at 1 and 10 mg/mL concentrations (Figure S10). For 50 mg/mL, the cell viability decreased slightly compared to the dark conditions, reaching 80%. Since cell viability is above 70%, which is the threshold specified in ISO standards,^55,56^ CS@ErB is biocompatible even at this high concentration. It should be noted that although ErB (FD & C red no. 3) is a food dye approved by the US Food and Drug Administration,^57,58^ high doses can cause side effects such as allergic reactions in the eyes, irritation of the skin,^59^ chromosome aberrations,^60^ and hyperactivity disorders.^61^

### PUF Performance Analysis

**Figure 5:**
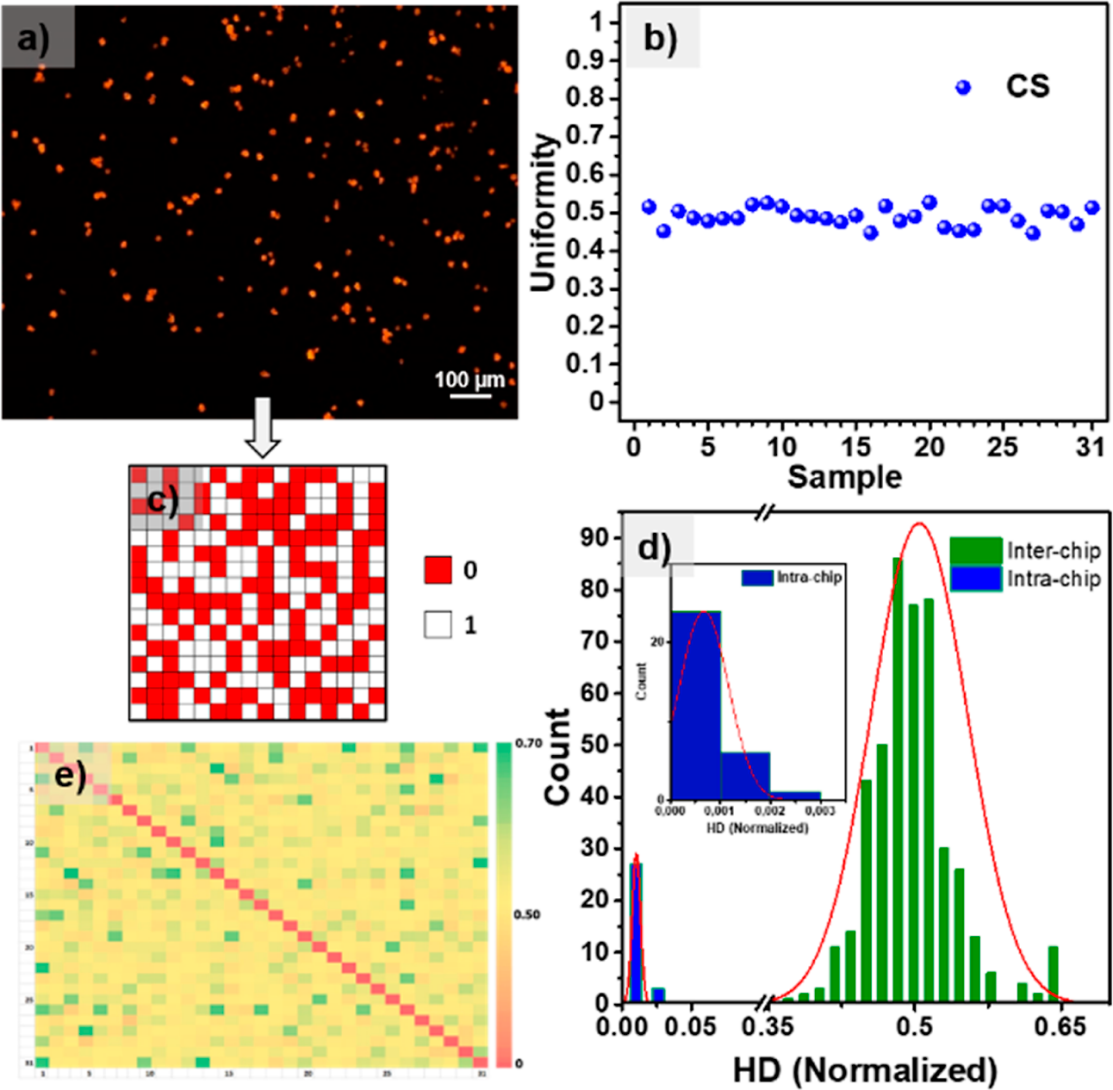
Key generation and extraction of PUF parameters. (a) Representative fluorescence microscopy image of the sample used in the PUF analysis. (b) Uniformity of bits obtained from 31 different PUF keys. (c) Representative binary key. (d) Distributions of HD_intra_ and HD_inter_ are presented on the left and right, respectively. (e) Pairwise comparison of HD_inter_ values.

To analyze the performance of the food-grade PUFs, we first converted fluorescence images to binary keys. The conventional method of PUF performance analysis involves the calculation of uniqueness, reliability, uniformity, and randomness.^62^ Fluorescence images of CS@ErB particles transferred onto gelatin were used for the PUF performance analysis. The authentication can be performed by taking fluorescence microscope images at regions designated by physical markers (Figure S11). These images were then processed to generate binary keys (Supporting Information, Table S1). A representative fluorescence microscopy image is presented in Figure 5a. This image size has been reduced to 16 × 16 pixels, leading to 256 bit long keys (Figure 5c). Red colors (0 bits) in the binary image correspond to CS@ErB, and white colors (1 bit) correspond to dark areas. Binary codes and security keys were generated for 31 PUFs (Supporting Information, Figure S12). First, the uniformity is calculated for each of these keys. Uniformity is a measure of the even distribution of 1 and 0 bits with an ideal value of 0.5.^31,62^ The raw response of physical systems can be uneven, with the degree of imbalance depending on the process’s physical characteristics. In other words, the raw key could have an unbalanced proportion of bits. One approach to addressing this issue is classic von Neumann debiasing. After this debiasing method was implemented, the uniformity of the fabricated PUFs improved significantly. The arithmetic mean of the uniformity of keys is 0.488, very close to the ideal value (Figure 5b). Another criterion, which is closely related to uniformity and also expresses the stochastic distribution of features, is randomness.^63^ To verify the randomness of food-grade PUFs, seven different NIST statistical tests were conducted using 62 sequences, each containing 128 bits. These sequences were obtained from a total of 31 different 256 bit long keys, resulting in a collection of 7936 bits for testing purposes. As a result, the p-value is greater than 0.01, and as evidenced by the minimum pass rate achieved, the generated bitstreams have successfully passed all the tests (Supporting Information, Table S2). Finally, the uniqueness and reliability of the PUFs were calculated. Uniqueness investigates the ability to distinguish one PUF from others and is calculated based on the interchip Hamming distance.^31,62^ Also, reliability is a measure of the repeatability of responses obtained from a PUF key under different conditions. We calculated the intrachip (HD_intra_) and interchip Hamming (HD_inter_) distance values in Figure 5d. HD_intra_ was calculated from images obtained from 31 different PUFs under five different lighting conditions for each PUF (Figure S13). The average HD_intra_ calculated from 31 different PUF keys exhibits a Gaussian distribution with an average value of 0.0006, very close to the ideal value of 0. The calculated mean HD_inter_ value shows a 0.50 center distribution. The intra- and interdevice distributions in Figure 5d do not overlap, indicating extremely low false positive and negative rates. The false positive and false negative rates obtained with the 0.180400 cutoff threshold were calculated to be 9.62 × 10^–13^ and 3.09 × 10^–12^, respectively. In addition, a pairwise comparison map of HD_inter_ values between chips is given in Figure 5e. Considering the uniqueness, reliability, uniformity, and randomness, the proposed approach is effective in the preparation of PUFs by using all food-grade materials.

**Figure 6:**
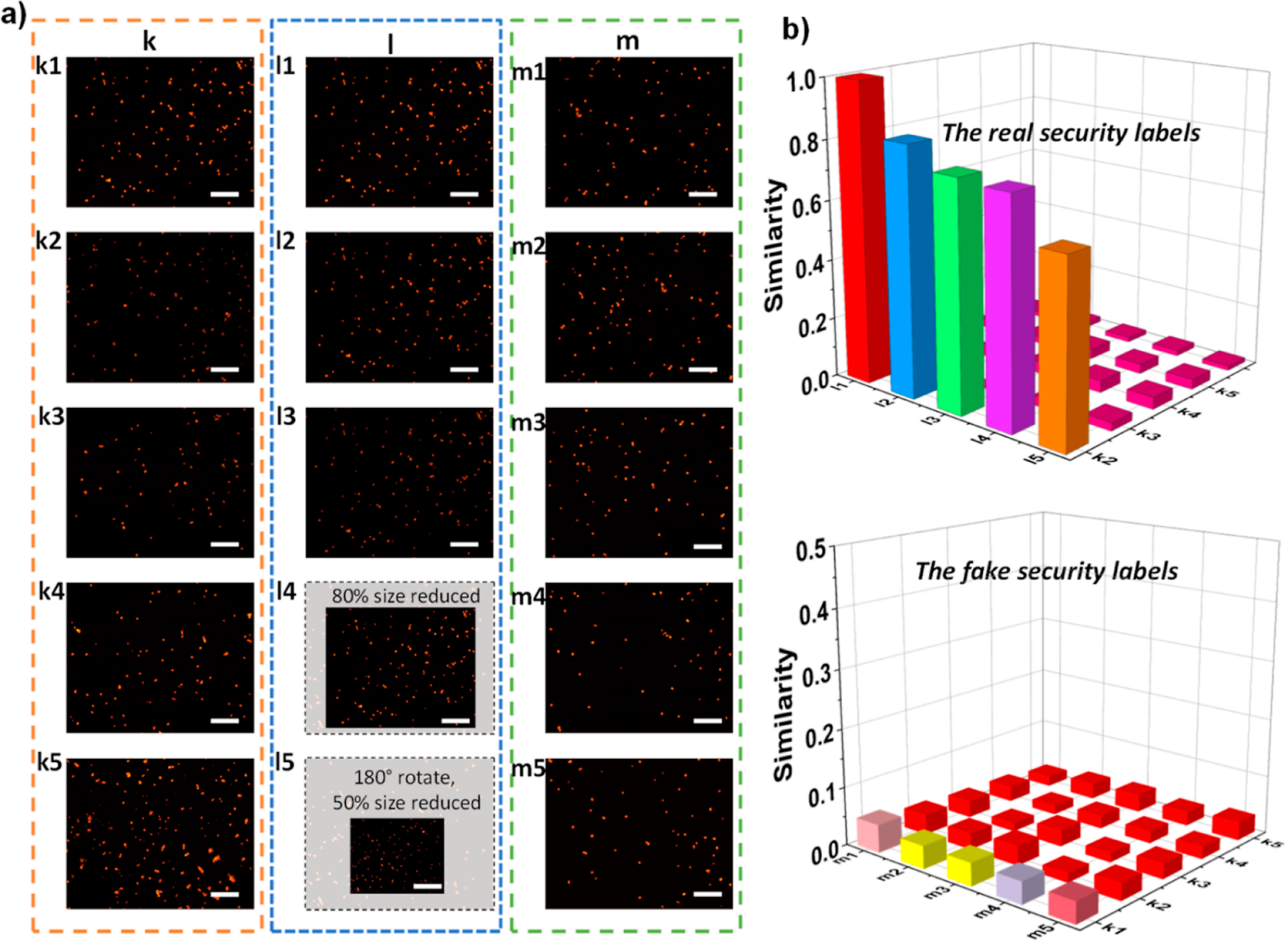
Authentication of PUFs was performed using the feature detection algorithm. (a) Fluorescence microscopy images. (b) Similarity values for various cases. (k1–k5) A database of fluorescence images captured from real security labels. (l1–l5) Fluorescence images taken from a real sample (k1) under various conditions (l1: recaptured, l2,l3: captured under different illumination, l4: captured under different magnification and illumination, and l5: captured under different magnification, illumination, and rotation). (m1–m5) Fluorescence images of fake labels. Scale bars are 100 μm.

Unclonable features can also be directly authenticated by using feature detection algorithms without the need for binary key generation. This approach offers several advantages, including fast processing, marker-less authentication, and the ability to authenticate complex features.^34,64–67^ PUF labels were imaged under varied illumination, rotation, and magnification conditions (Figure 6). The feature detection algorithms oriented FAST and rotated BRIEF (ORB)^68,69^ were used to identify images. Similarity (*S*) among the PUF labels was determined by using the following equation^66,67,70^
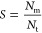
1where *N*_m_ represents the number of matching features, whereas *N*_t_ represents the total number of features. The similarity values for genuine labels (k1 between l1–l5) are depicted in Figure 6, showing *S* values of 1, 0.83, 0.76, 0.75, and 0.60 for l1 (second capture), l2, l3 (captured under different illumination), l4 (captured under different magnification and illumination), and l5 (captured under different illumination and rotation), respectively. Conversely, the similarity of fake labels (m1–m5) compared to genuine labels (k1–k5) is less than 0.05. These results obtained through feature-matching analysis provide practical implications. The significant difference in similarity values between genuine and fake labels allows for the determination of a suitable threshold value, ensuring a wide range of authenticity even in images captured under diverse conditions.

Another important metric for security labels is the encoding capacity, which refers to the maximum number of unique PUFs that can be constructed.^71–73^ Ideally, when the encoding capacity is defined as *C*^s^, the value of *C* is 2 for binary bits 0 and 1, and the value of sis the key size. Many PUF systems have estimated their encoding capacity by taking into account independent bit elements in bit sequences known as degrees of freedom (DoF). Based on the DoF (see the Supporting Information for details) calculation, the encoding capacity is found to be 2^103^. The encoding capacity can be further increased by using multiple challenge–response pairs. Initial results show great promise in generating multicolor samples by using green-fluorescent molecule-doped CS particles (Figure S14).

### Stability of PUFs

**Figure 7:**
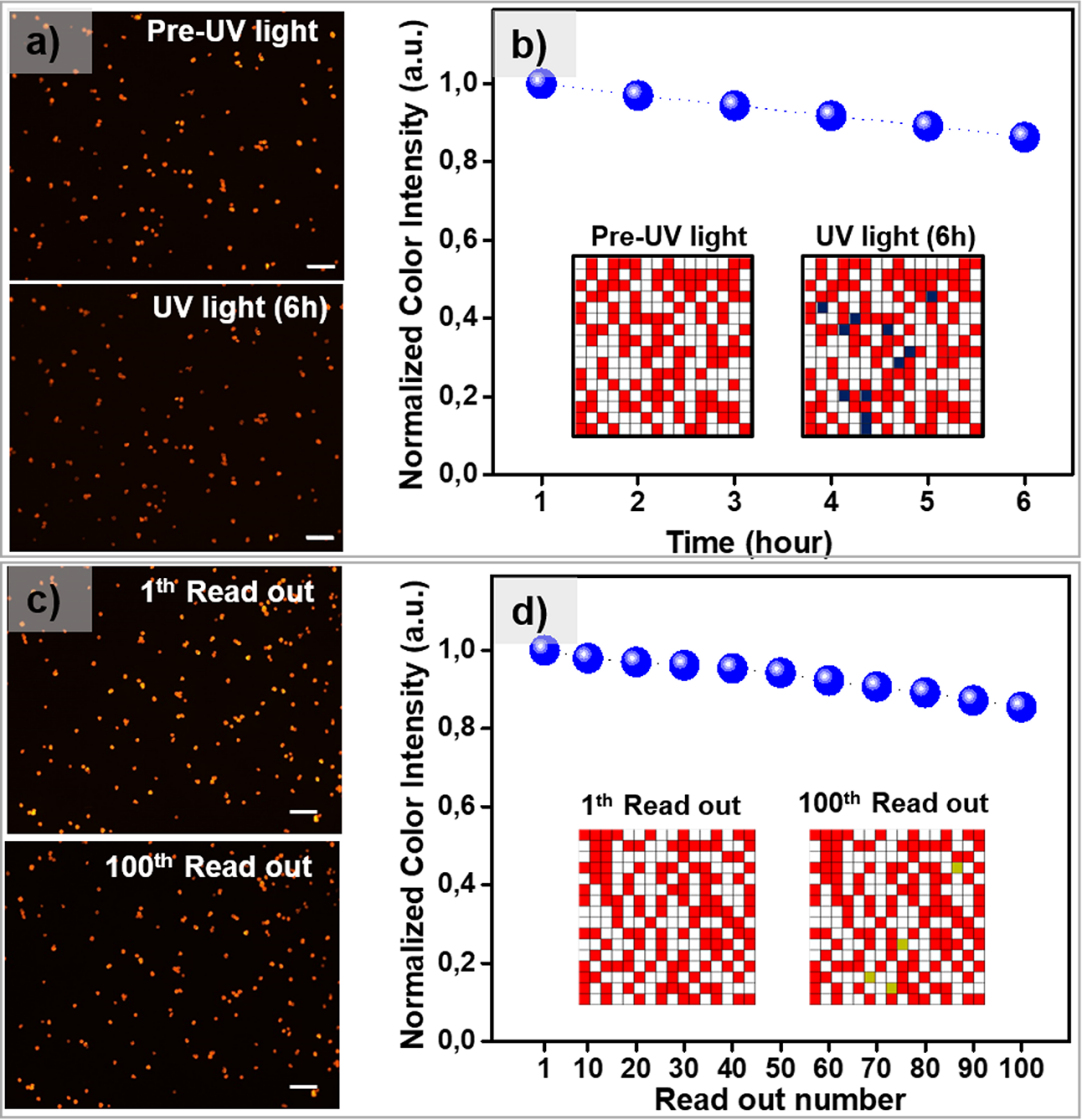
Stability of PUFs. (a) Photostability of PUFs under exposure to UV light. Fluorescence microscopy images of the sample before (top) and after 6 h of UV light exposure (down). (b) Fluorescence intensity as a function of the duration of UV light exposure using a source with a power of 3 W at a wavelength of 365 nm. The inset shows the binary keys before (left) and after (right) the UV light test. (c) First (up) and 100th (down) fluorescence microscopy images were taken at 1 min intervals from the same region of a PUF sample. (d) Fluorescence intensity as a function of the number of readouts. The inside shows the binary keys of the 1st and 100th readout. Scale bars are 100 μm, and the exposure time is 600 ms.

The stability of PUFs was studied by using different techniques. To determine the photostability of fluorescent PUFs produced with CS@ErB particles, three different photostability tests were performed (Figure 7). The stability was first studied under UV light exposure. Qualitatively, there was no significant change in the fluorescence microscopy images before and after exposure to UV light for 6 h (Figure 7a). The fluorescence intensity showed a slow decay under UV light and was 86.3% of the initial intensity after 6 h (Figure 7b). The similarity rate of binary keys derived from fluorescence images taken before and after UV light exposure is 95.70%. An important consideration is the read-out stability. Ideally, a PUF should be successfully authenticated repeatedly. To test the read-out stability, 100 images were taken from the same region of the PUF sample at 1 min intervals (Figure 7c). After taking 100 consecutive images, the fluorescence intensity was 85.5% of the initial intensity (Figure 7d). The similarity rate of binary keys extracted after the first and 100th readings is 98.4%. In an additional experiment, the fluorescence microscope images were taken after ambient storage of PUFs for 4 months. There was no discernible change after ∼4 months of storage (Figure S15). Note that the randomly positioned starch particles are also visible in optical microscopy under a bright field. The stability of these particles greatly exceeds that of the fluorescent molecule. In another stability experiment, fluorescence images were captured from a specific region of the PUF under identical conditions before and after a 24 h exposure to daylight. The images, presented in Figure S16, demonstrate that there were no significant changes observed in the fluorescence emission of the PUF samples. The absence of noticeable alterations in the fluorescence emission suggests that the PUF labels possess excellent light stability, making them suitable for long-term applications in which exposure to ambient light is inevitable. In addition to the various light exposure tests, the physical integrity and emission properties of the PUF labels were successfully maintained during a mechanical abrasion test. The PUF labels were subjected to mechanical rubbing and abrasion, and the results, depicted in Figure S17, indicate that the labels retained their physical integrity and emission characteristics without significant changes. The combination of excellent light stability and resistance to mechanical abrasion enhances the overall reliability and longevity of the PUF labels, making them a promising solution for a wide range of practical applications where durability and performance are crucial factors. For all stability tests, binary keys extracted from fluorescence images of samples before and after the stability tests were compared (Figures 7b,d and S15–S17). Notably, the similarity in the key sequences remains remarkably high (>96%). These results showcase that PUFs subjected to different conditions possess a stable response for authentication.

## Conclusions

In conclusion, this study has presented a practical route to security labels based on all food-grade materials. At the center of the approach are microscale starch particles derived from corn, which is a common ingredient in food products. The proper length scale and adsorbent characteristics make CS particles a highly viable building block for security labels that rely on physically random features. The adsorption of a food colorant, ErB, results in luminescent CS@ErB particles. The random deposition of these particles is facilitated by spin-coating at low concentrations. The fabricated PUFs exhibit appealing characteristics in terms of PUF performance metrics. The excellent biocompatibility of PUFs emerges from food-grade materials. This approach is particularly suitable for on-dose and on-food security labels for anticounterfeiting applications. Furthermore, the presented PUFs are cost-effective as they are composed entirely of food-grade materials. The estimated cost per security label is less than $0.1 (see the Supporting Information for details). The presented approach can contribute to food security in relation to the Sustainable Development Goal 2 set by the United Nations. Future work based on CS particles appears to be promising in different directions that range from the use of other taggants together with different manufacturing routes and encapsulation layers for application on nonplanar substrates such as curved pills.

## Supporting Information Available

Photoluminescence spectra, optical microscopy, electron microscopy, and Raman mapping images, EDX, TGA, and XRD analysis, additional data for biocompatibility tests, details regarding the process of key extraction and calculation of PUF metrics, additional data for PUF performance, stability tests, and cost analysis (PDF)
Video of application demonstration of food-grade edible PUF on drugs (MP4)
