# Supplementary material for: Food-Grade Physically Unclonable Functions
Source: ACS Appl Mater Interfaces. 2023 Aug 24;15(35):41373–84. doi: 10.1021/acsami.3c09035 (PMC10485800; doi:10.1021/acsami.3c09035)
Supplement: Supplementary file 1 — am3c09035_si_001.pdf [file am3c09035_si_001.pdf]

Supporting Information

Food-Grade Physically Unclonable Functions

Abidin Esidir[1,2], Nilgun Kayaci[1], N. Burak Kiremitler[1,2], Mustafa Kalay[1,3], Furkan Sahin[1,4], Gulay Sezer[5], Murat Kaya[6], M. Serdar Onses[1,2,*]*

[1] ERNAM - Nanotechnology Research and Application Center, Erciyes University, Kayseri, 38039, Turkey

[2] Department of Materials Science and Engineering, Erciyes University, Kayseri, 38039, Turkey

[3] Department of Electricity and Energy, Kayseri University, Kayseri, 38039, Turkey

[4] Department of Biomedical Engineering, Faculty of Engineering and Architecture, Beykent University, 34398 İstanbul, Turkey

[5] Department of Pharmacology, Erciyes University, Faculty of Medicine, 38039, Kayseri, Turkey

[6] Department of Molecular Biology and Genetics, Faculty of Science and Letters, Istanbul Technical University, 34469 Istanbul, Turkey

* Address correspondence to: onses@erciyes.edu.tr, nuriburak@erciyes.edu.tr

Number of pages: 22

Number of tables: 2

Number of figures: 17

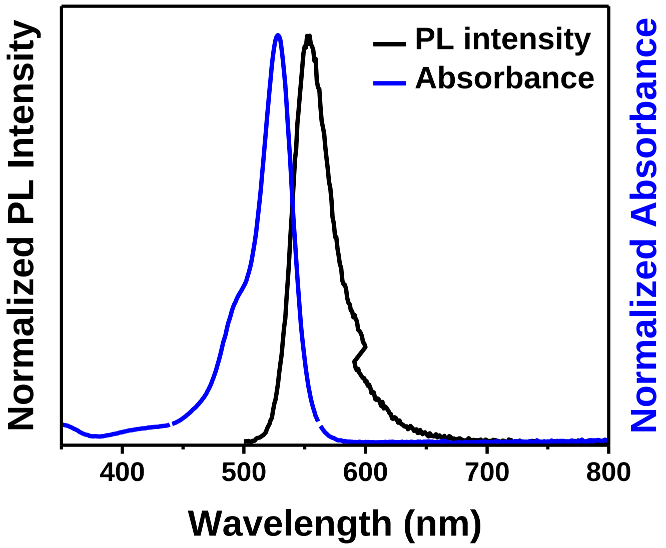

Figure S1. The photoluminescence spectrum (left) and absorbance spectrum (right) of erythrosine B.

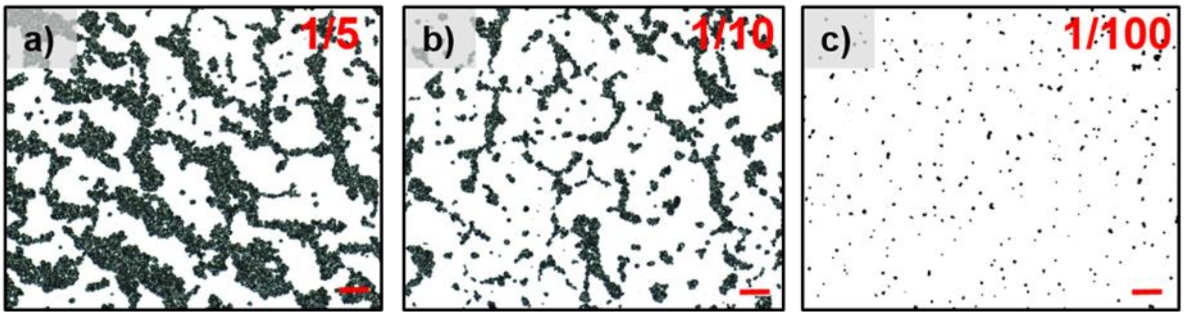

Figure S2. Optical microscopy images of the surfaces following the spin-coating of dispersions. The weight/volume ratio of corn starch to water a) 1/5, b) 1/10 and c) 1/100. Scale bars are 100 μm.

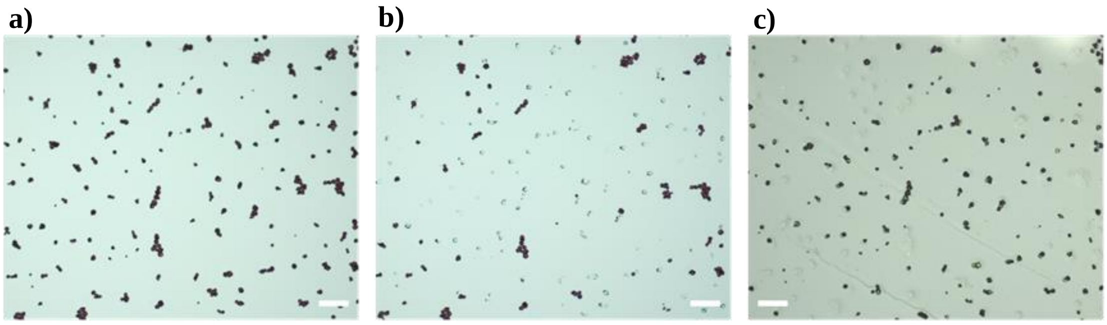

Figure S3 Optical microscope images of the substrates before and after the transfer process. a) The silicon substrate before the transfer process, b) Silicon substrate after transfer of the starch particles to the gelatin substrate. c) Gelatin substrate after the transfer process. Scale bars are 100 μm.

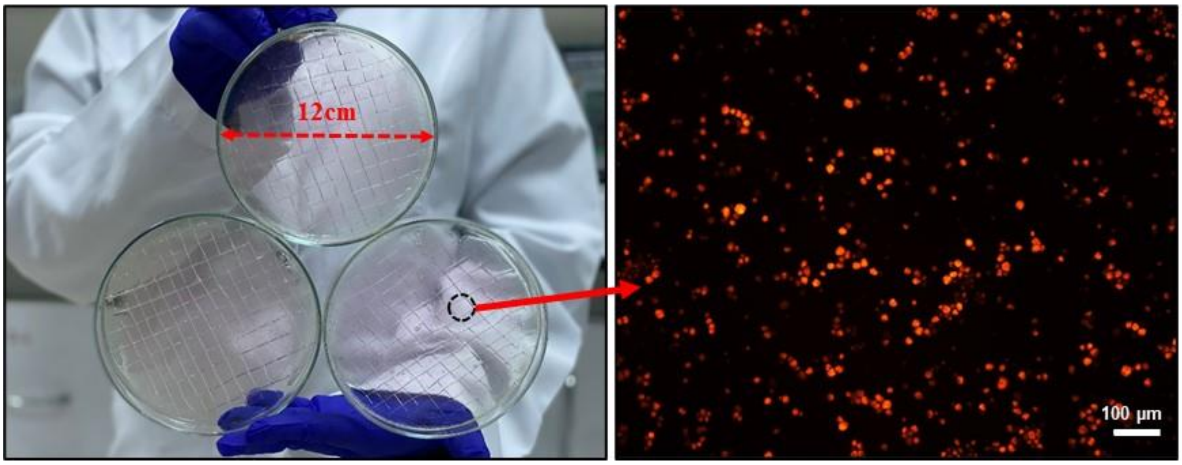

Figure S4. Demonstration of scalable production of PUFs. Photograph of PUF labels obtained by transfer of randomly positioned CS@ErB features from the 4-inch silicon wafer to gelatin (left), a fluorescence microscope image the sample is shown on the right.

a) b) c)

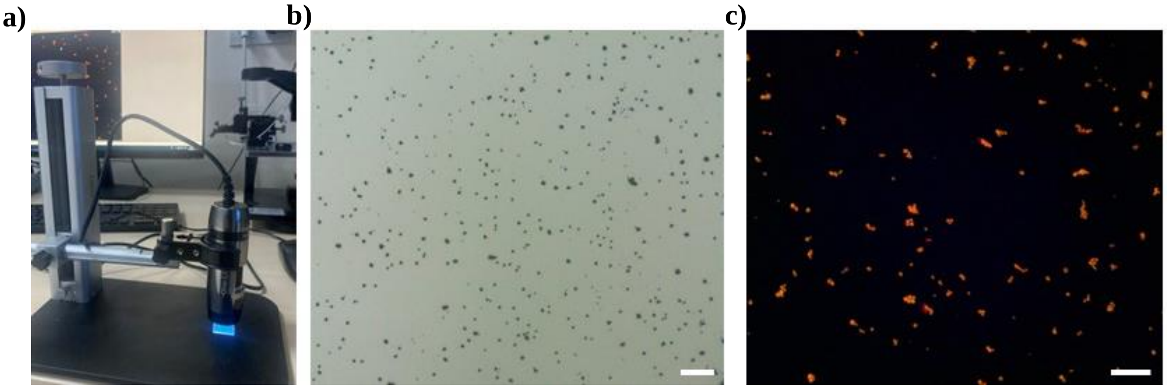

Figure S5. Imaging demonstration with a handheld microscope. a) Photograph of the handheld microscope (Dino-Lite). b) Bright field and c) fluorescence images acquired from PUF labels via the handheld microscope. Scale bars are 200 μm.

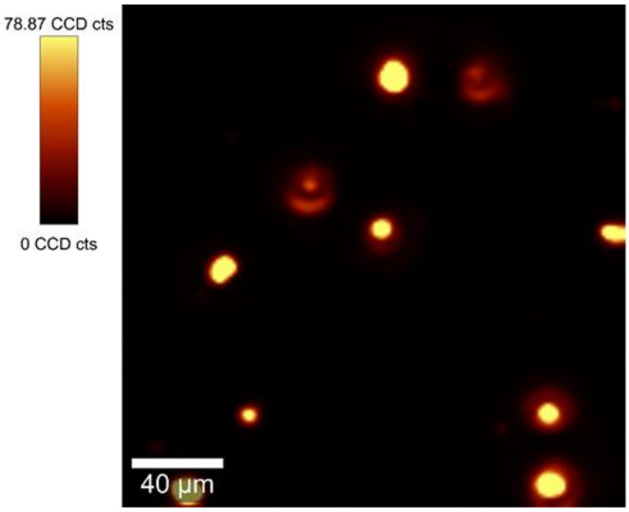

Figure S6. Raman mapping image of CS@ErB particles.

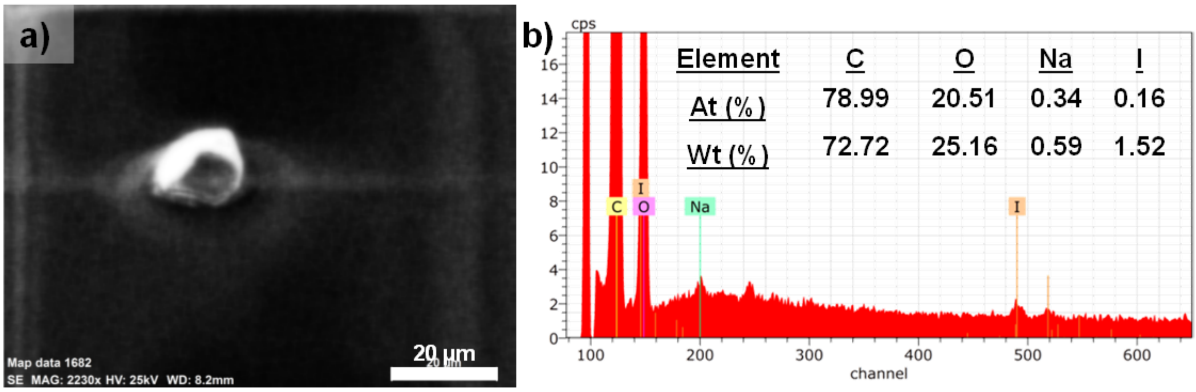

Figure S7. a) SEM image and b) EDX elemental analysis of the CS@ErB particles.

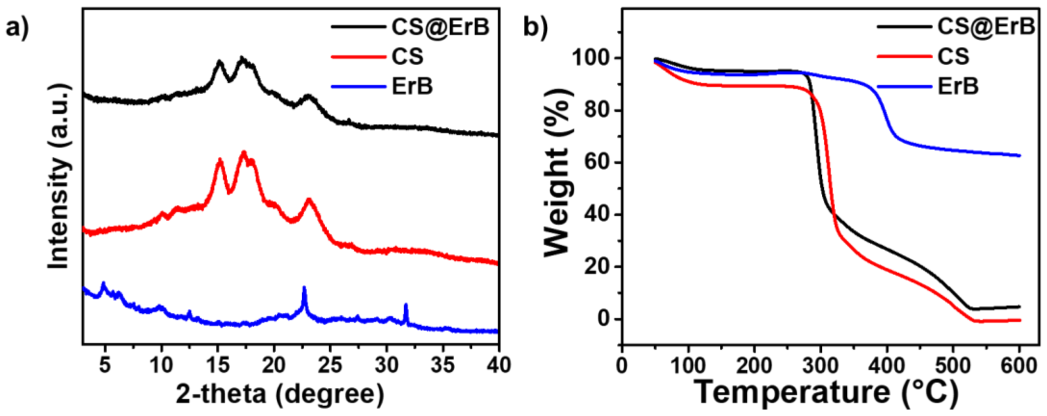

Figure S8. XRD patterns a) and TG curve b) of ErB, CS and CS@ErB.

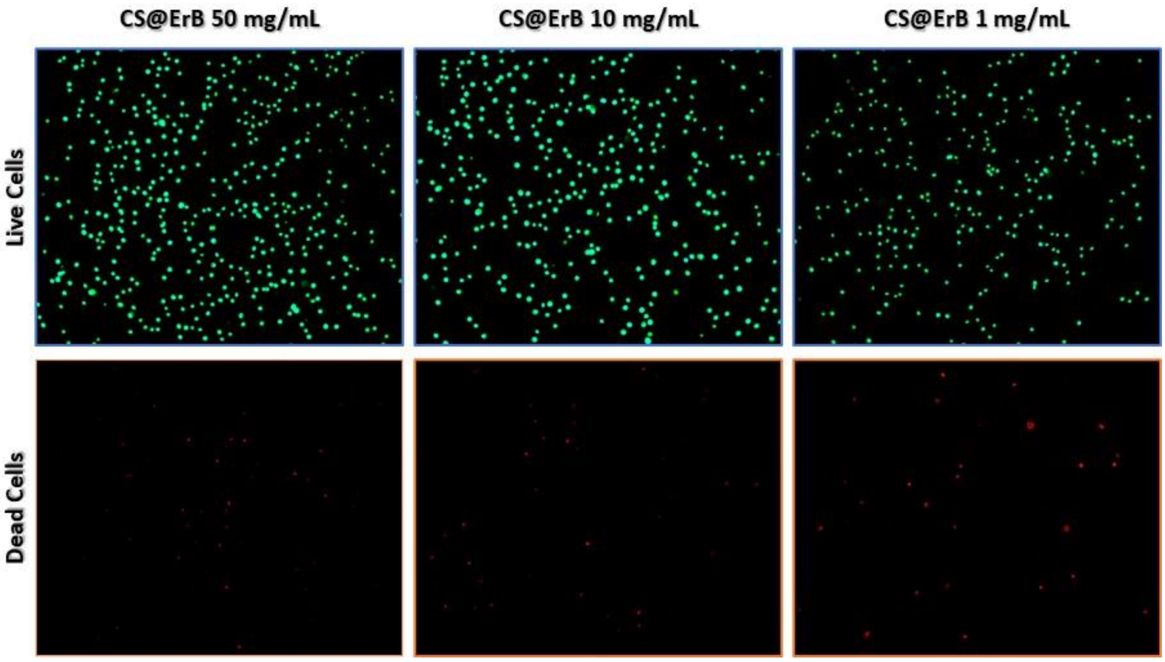

Figure S9. Fluorescence microscope images of live and dead cells. Green fluorescent cells are alive, whereas red cells are dead. For the images, the ratio of the number of damaged/dead cells to the total number of cells from left to right is 6.1%, 6.4%, and 7.9%, respectively. This rate is 5.8% for the control group.

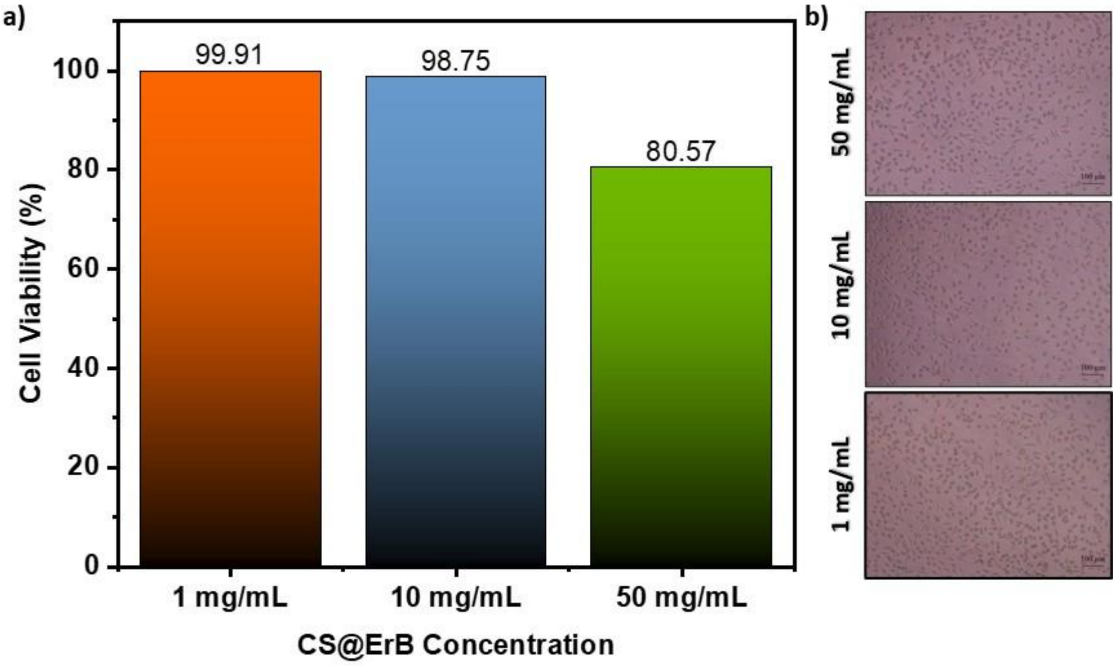

Figure S10. Biocompatibility against reactive oxygen species generated by light exposure as a function of CS@ErB concentration. a) Cell viability, b) optical microscope images.

Extraction of binary keys:

MATLAB was used to extract binary keys from images. The acquired images were decomposed by image processing. Each image was then used to create bitmaps. Von-Neumann de-biasing was applied. The threshold was applied on images with a pixel size of 2752×2208. The image size was then reduced to 16×16 pixels. This image was then digitized to obtain a 256-bit key consisting of 0-bits and 1-bits. This procedure was repeated for all images (Figure S2). To further verify the randomness of the extracted bits, National Institute of Standards and Technology (NIST) randomness test suite was used. The keys are considered random when the p-value ≥ 0.01.

Table S1. Key extraction steps

Original Image	Grayscale	Noise Reduction (Threshold)	Binary	Von-Neumann debiasing operation	Response bit extraction	Extracted Key
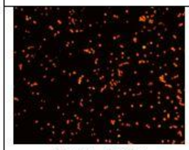<br>2752x2208	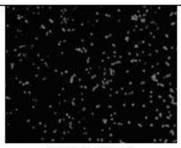<br>2752x2208	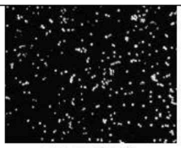<br>2752x2208	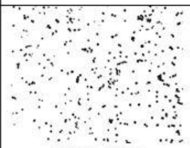<br>2752x2208	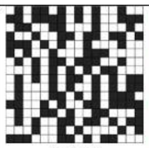<br>16x16<br><br>16x16-bit security key obtained from 0s and 1s after von-neumann debiasing.	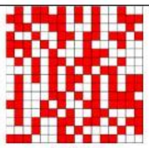<br>16x16<br>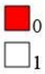	1010010101011011<br>0010000101011000<br>1001100111011101<br>0110110010010010<br>0001010110111011<br>0101101110001111<br>1010100100011011<br>1110101010100111<br>1010101001010100<br>1011101011010100<br>1011100101010001<br>0011010110010000<br>1011001011001110<br>1010110010010100<br>1110110101111010<br>0001110010100111

Uniformity

The uniformity for each key was calculated with the following formula.

$$\text{Uniformity} = \frac{1}{n}\sum_{i=1}^{n} r_{i,l} \ \text{x}100\%$$

n: the number of bits

rl: lth binary bit (0 or 1) of an n-bit response from a key

Uniqueness

Uniqueness was calculated using 31 keys obtained from the images presented in Figure S11.

$$\text{Uniqueness} = \frac{2}{s(s-1)}\sum_{i=1}^{s-1}\sum_{j=i+1}^{s}\frac{HD(R_i,R_j)}{n}$$

S: the number of keys from different chips

HD (R_i, R_j) : Hamming distance between chips i and j

Normalized Hamming distance = Hamming distance divided by the number of bits, $\frac{HD(R_i,R_j)}{n}$

In the uniqueness formula, the sum of normalized Hamming distances is divided by the total number of comparisons of among different chips, $\frac{S(s-1)}{2} = \frac{31(30)}{2} = 465$ in our case.

Reliability

Reliability tells us how reproducible a PUF is in reproducing bits.

$HD_{INTRA} = \frac{1}{m}\sum_{t=1}^{m}\frac{HD(R_i,R'_{i,t})}{n}$

m: number of samples studied under different conditions

Reliability = 1.00-HD_{INTRA}

Degree of Freedom (DoF)

$$DoF = \frac{\mu(1-\mu)}{\sigma^2}$$

DoF = l.

μ: The HD mean value for different samples

σ: the SD of the different samples

Classic von Neumann Debiasing

The following procedure was used for debiasing

- Consider the key is composed of consecutive pairs of bits
- Discard the pair of bits in the case of bits consisting of 11 or 00
- Retain the first bit, in the case of bits consisting of 10 or 01

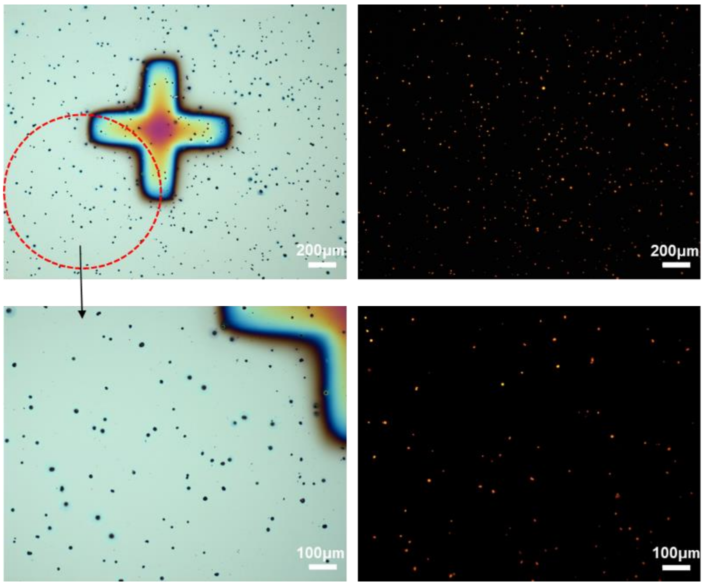

Figure S11. The definition of markers to determine the exact location of the regions, where optical and fluorescence microscope images are taken. The marker was fabricated by physical vapor deposition of ZnO through a stencil mask.

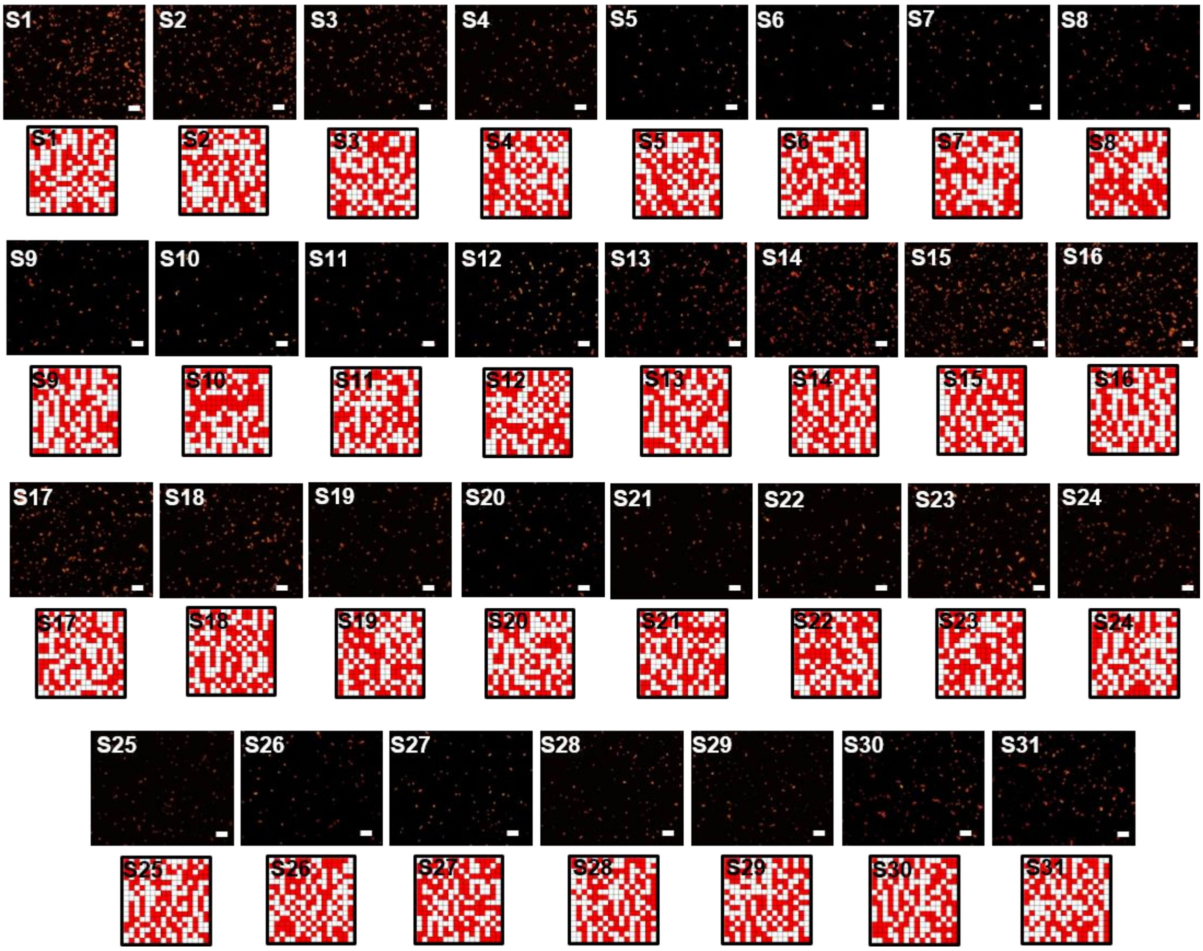

Figure S12. Florescence microscopy images and corresponding 16×16 keys of different samples fabricated using CS@ErB. Scale bars are 100 μm.

Table S2. The NIST randomness test results for keys generated from food-grade PUFs.

NIST statistical test	*n*	*p*-value	Pass rate	*M* or *m*	# of sub-test	Result
Frequency	128	0.719152	60/62	-	1	Pass
Block frequency		0.764676	61/62	20	1	Pass
Cumulative sums		0.698100<br>0.819889	60/62<br>60/62	-	2	Pass
Runs		0.196094	61/62	-	1	Pass
Longest run of ones		0.214623	62/62	8	1	Pass
Approximate entropy		0.301516	60/62	2	1	Pass
Serial		0.980224<br>0.966282	62/62<br>62/62	4	2	Pass

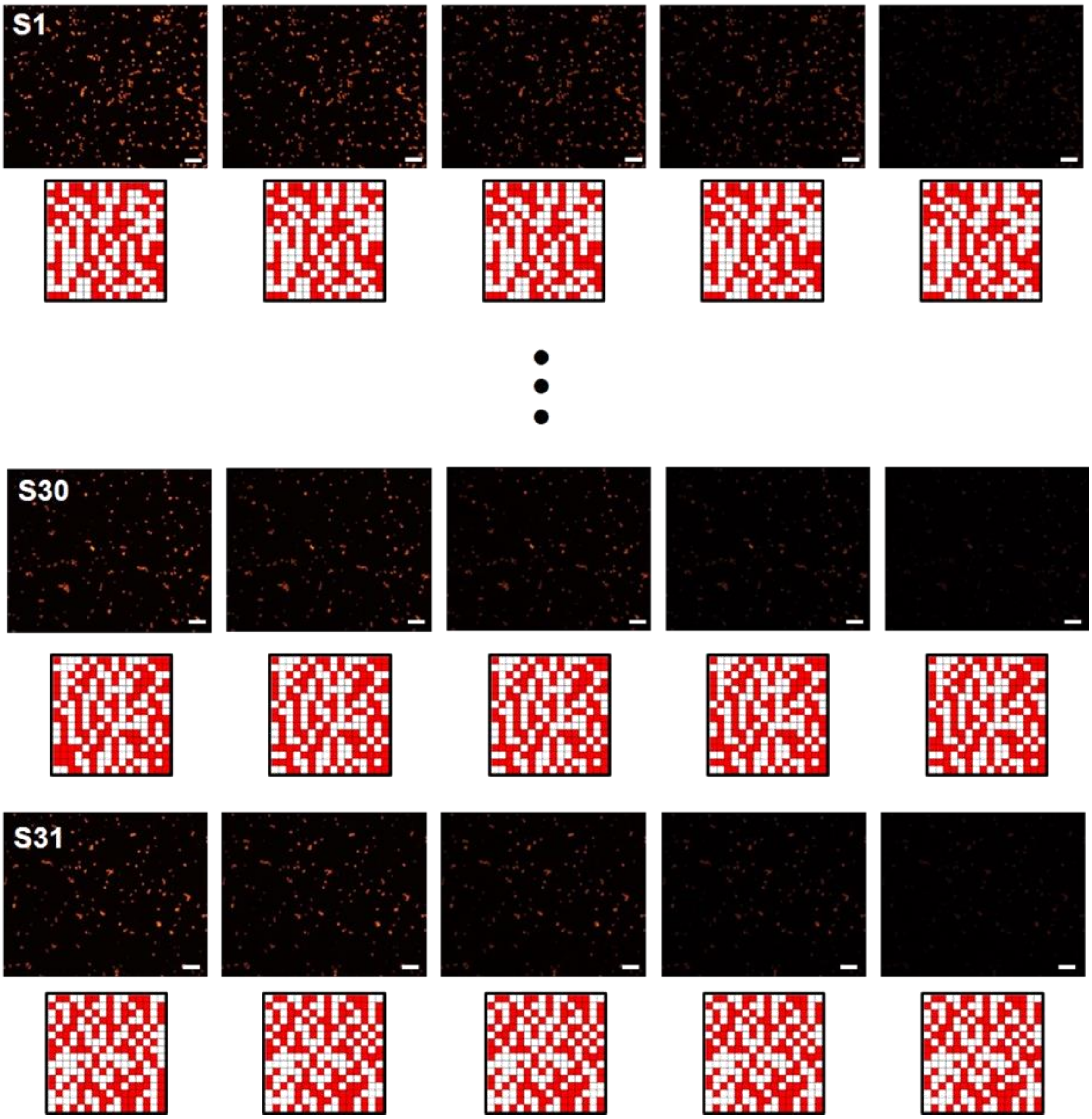

Figure S13. Representative images acquired from a selected region by varying illumination conditions: exposure in the range of 250 ms to 600 ms, gamma value 1. The keys extracted from these images were used in the calculation of HD_{INTRA}. Scale bars are 100 μm

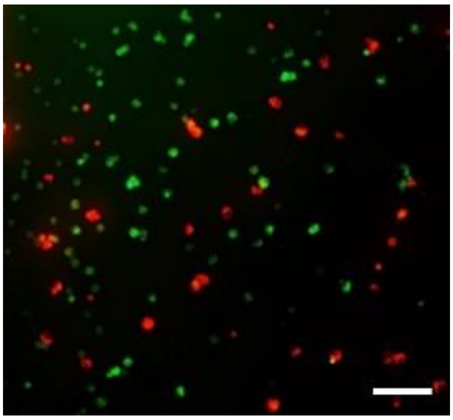

Figure S14. Fluorescence image of multicolor/spectral PUF containing CS@ErB and CS@Curcumin particles. Scale bar is 100 μm.

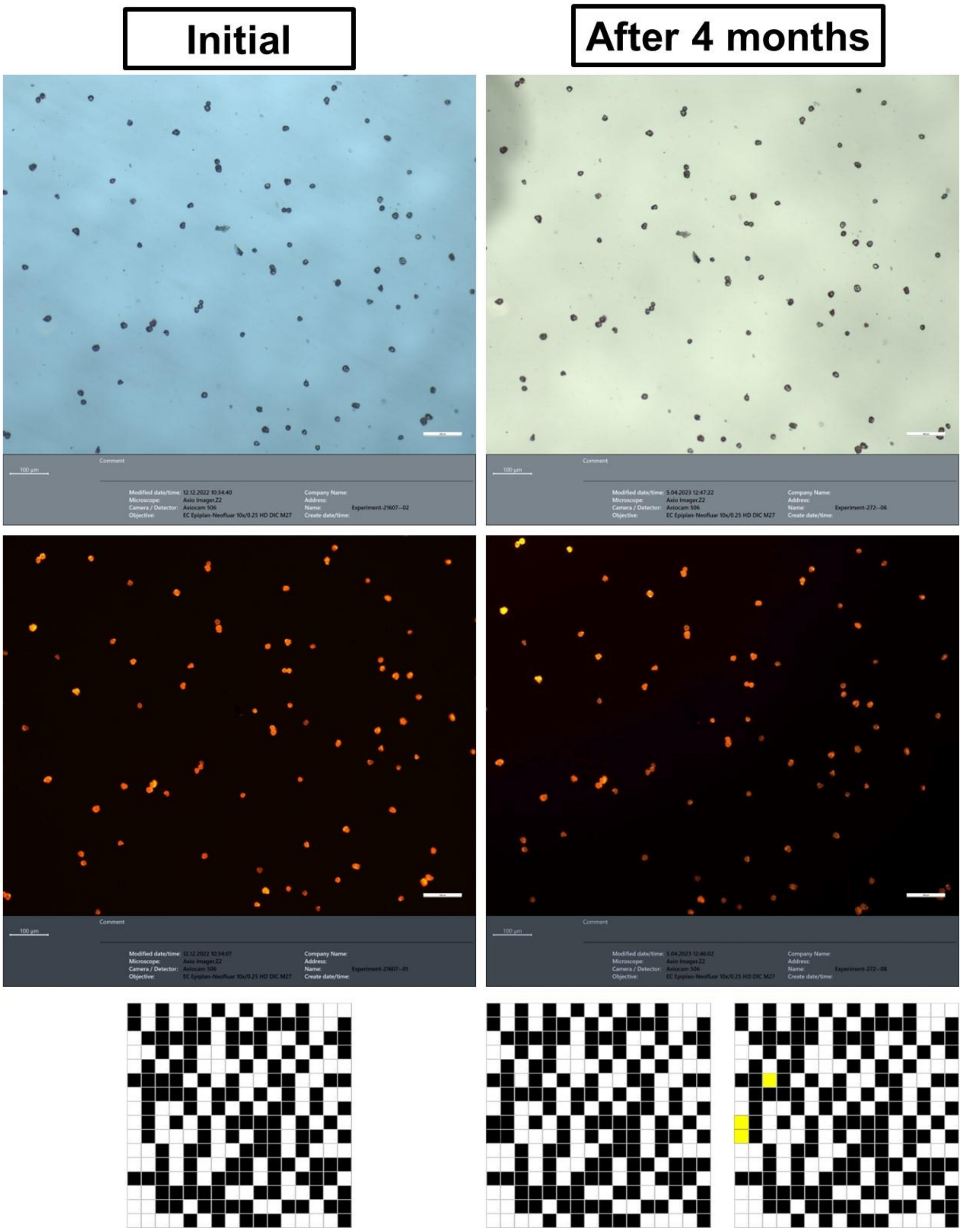

Figure S15. Long-term stability test of a PUF sample. Bright field, fluorescence images and corresponding binary keys extracted from fluorescence images of a sample stored for 4 months. Data zones of the images have been intentionally left to indicate the elapsed time. Fluorescence images taken at the same exposure time (600 ms). The similarity ratio between the bit sequences generated before and after the test is 98.8%.

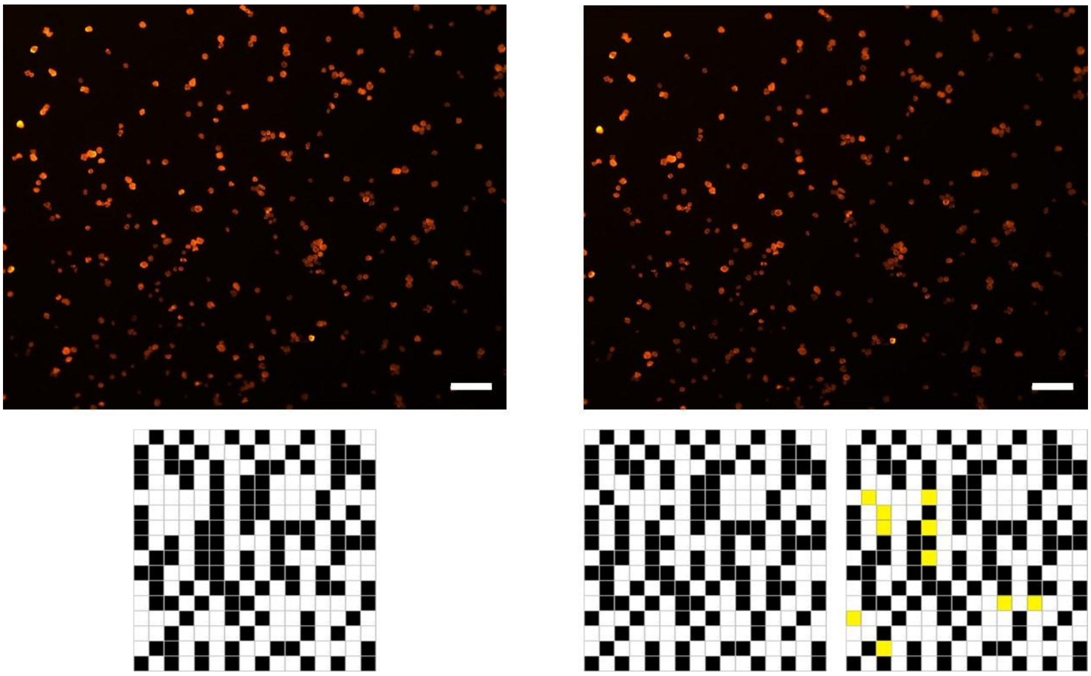

Figure S16. Daylight stability of PUFs. Fluorescence microscope images corresponding extracted binary keys before (left) and after 1 day exposure to day light. Scale bars are 100 μm. The similarity ratio between the keys before and after the test is 96%.

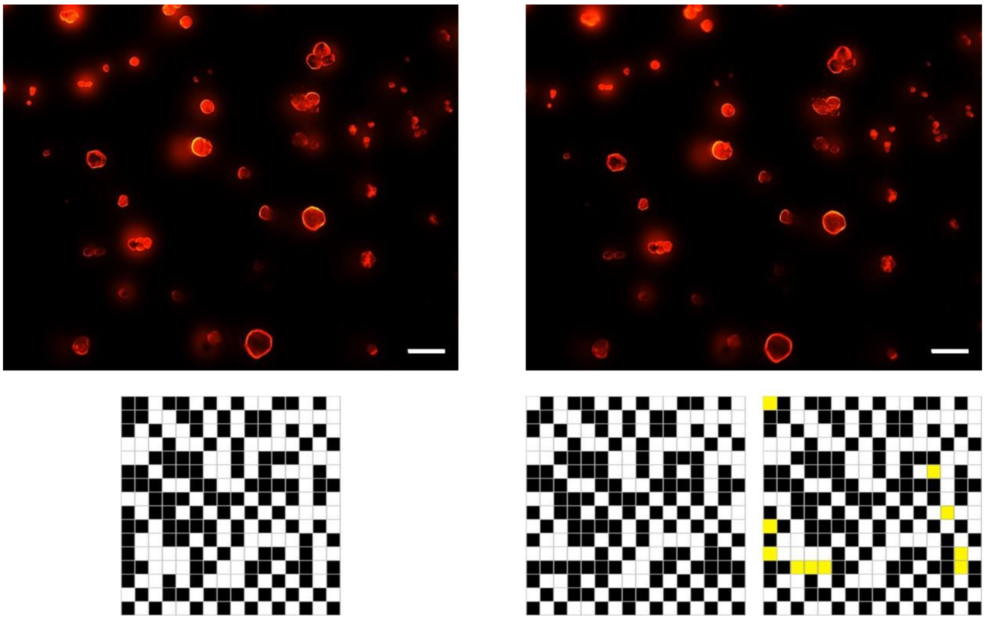

Figure S17. Abrasion stability of PUFs. Fluorescence microscope images and corresponding binary keys before (left) and after the abrasion test. Specifically, the sample was glued under the weight of 200 g and moved 30 cm against an aluminum foil. Scale bars are 50 µm. The similarity ratio between the keys before and after the test is 96%.

<u>Cost analysis of the Food-Grade PUFs:</u>

A cost analysis was performed on a material basis for PUFs fabricated on gelatin films measuring 1×1 cm^2 each. A gelatin solution of 8 g was prepared in 60 mL, and gelatin films were obtained by pouring 8 mL of the solution into petri dishes with a diameter of 5.8 cm. From each petri dish, 16 gelatin films of 1x1 cm^2 were derived. Therefore, the cost of the gelatin film consumed per PUF label can be calculated as follows:

The cost per gelatin substrate: (10 g / 60 mL) × (8 mL / 16 pieces) × ($0.057/g) = $0.083/piece

For each gelatin substrate, a 50 μL solution of CS@ErB, coated on a silicon surface, was used. ErB had a concentration of 5 mM, and CS had a concentration of 0.1 g/mL. Therefore, the cost of CS and ErB consumed per each gelatin substrate can be calculated as follows:

For ErB: (0.005 mol/L) × (10^{-6} L/50 μL) × (882 g/mol) × ($0.2564/g) = $2.26 .$10^{-7}$/piece

For CS: (0.1 g/1000 μL) × (50 μL/piece) × ($0.0043/g) = $2.15 × 10^{-5}/piece

The total cost: $0.083 + $2.26 .$10^{-7}$ + $2.15 × 10^{-5} = ~$0.083/per PUF label.

The CS and gelatin utilized in this cost analysis were obtained in small amounts. Cost reductions are expected upon scaling up the production.
